# Ethnic differences in folic acid supplement use in a population-based cohort of pregnant women in Norway

**DOI:** 10.1186/s12884-017-1292-x

**Published:** 2017-05-15

**Authors:** Tarja I. Kinnunen, Line Sletner, Christine Sommer, Martine C. Post, Anne Karen Jenum

**Affiliations:** 1 Faculty of Social Sciences /Health Sciences, University of Tampere, Tampere, Finland; 2 Department of Child and Adolescents Medicine, Akershus University Hospital, Lørenskog, Norway; 3 Department of Endocrinology, Morbid Obesity and Preventive Medicine, Oslo University Hospital, Oslo, Norway; 4 Health Agency, City of Oslo, Oslo, Norway; 5 Institute of Health and Society, Department of General Practice, Faculty of Medicine, University of Oslo, Oslo, Norway

**Keywords:** Ethnicity, Pregnancy, Folic acid, Dietary supplements, Education, Unplanned pregnancy

## Abstract

**Background:**

Peri-conceptional use of folic acid supplements is recommended to prevent neural tube defects. Correct supplement use seems to be less common among ethnic minorities. We examined ethnic differences in folic acid supplement use before and during pregnancy and possible effect modification by education or planning of pregnancy.

**Methods:**

The participants were 811 healthy pregnant women from a population-based cohort study in Oslo, Norway in 2008–2010. Ethnicity was categorized to five groups (European, Middle Eastern, South Asian, East Asian, African). Data on folic acid supplement use were obtained from hospital records and remaining data by a questionnaire. Logistic regression analyses were adjusted for age, parity, planning of pregnancy, education and Norwegian language skills.

**Results:**

Before pregnancy, 30.1% of European women and 7.1 to 13.6% of women in the other ethnic groups used folic acid supplements (*p* < 0.001). The adjusted odds ratio (OR) for supplement use was 0.55 (95% confidence interval 0.31; 0.96) for South Asian and 0.42 (95% confidence interval 0.19; 0.94) for Middle Eastern women compared with European women. During pregnancy, supplement use was most common in European women (65.7%) and least common in Middle Eastern (29.4%) and African women (29.0%) (*p* < 0.001). Compared with European women, all other ethnic groups had lower adjusted odds (OR 0.30 to 0.50, *p* < 0.05 for all) for supplement use among women with high school or less education, but not among more educated women. Planning of pregnancy did not modify the association between ethnicity and supplement use.

**Conclusions:**

Few women used folic acid supplements before pregnancy. Educational level modified the association between ethnicity and supplement use during pregnancy. Public health campaigns should focus on increasing awareness especially in ethnic minority groups with low educational level.

## Background

Neural tube defects such as spina bifida and anencephaly are among the most common type of serious birth defects, occurring when the neural tube fails to close during the first weeks of gestation [1, 2]. The overall prevalence of neural tube defects was 9.1 per 10.000 births in Europe and has not decreased since 1991 [3]. In Norway, 9.3 per 10.000 births were affected by a neural tube defect in 1991–2011 [3]. Folic acid supplementation has been shown to reduce the risk for neural tube defects remarkably [4] and it may also have other health benefits [5–8].

Recommendations for peri-conceptional folic acid supplementation vary in Europe [9]. In Norway, all women planning a pregnancy or who may become pregnant, are recommended to use folic acid supplements (0.4 mg/day) at least from one month before conception until three months gestation [10]. It is important to start supplementation weeks before conception to achieve adequate folate status by the first few weeks after conception. However, a cohort including 22500 ethnic Norwegian women from years 2000–2003 showed that only 12% of the women used the supplements before pregnancy and 70% at some point during pregnancy [11]. A population-based cross-sectional study from Oslo, Norway, reported that 17% of women had started using the supplements before pregnancy and 58% had used them at some point during pregnancy [12]. Another alarming finding in this study was that non-Western immigrant women were clearly less likely to use the supplements than Western women (2 vs. 22% before pregnancy, 19 vs. 73% during pregnancy, respectively). However, the study did not adjust for important confounders such as planning of pregnancy or education, and categorization of ethnicity was very crude.

Ethnic differences in folic acid supplement use before and/or during pregnancy have been studied in a few other European countries (Netherlands, Belgium, Ireland and the United Kingdom) [13–20]. While these studies included various ethnic groups, the main findings were similar showing that supplement use was less common among most ethnic minority groups than among the comparison groups. However, which and how many variables were controlled for in the analyses varied substantially between the studies. In these studies and the Norwegian study [11], various other factors such as low education, low socio-economic status, poor skills in local language, young age, unplanned pregnancy, multiparity and late booking in antenatal care were identified as risk factors for not using folic acid supplements before or during pregnancy. None of these studies reported if these risk factors modified the relationship between ethnicity and supplement use, which might have significant implications for implementation of the findings. Identifying possible effect modifiers helps to target public health messages and interventions for the most vulnerable subgroups not using the supplements.

The aim of the present study was to describe ethnic differences in folic acid supplement use before and during pregnancy in a population-based cohort of pregnant women in Oslo, Norway, and whether possible differences were modified by education or planning of pregnancy.

## Methods

### Study design and population

The study was originally set up to investigate predictors of gestational diabetes mellitus and fetal growth. The methods of this population-based prospective cohort study have been described in detail previously [21]. The study population included pregnant women who attended primary care Child Health Clinics for antenatal care in Groruddalen, in Oslo, Norway. A large proportion of the population in this area has ethnic minority background and 75 to 85% of pregnant women utilize the antenatal services of these clinics. The population represents the main ethnic groups living in Oslo.

To be eligible for the study, women had to live in one of three city districts in Groruddalen, plan to give birth at one of the two study hospitals, be at ≤20 weeks’ gestation, be able to communicate in Norwegian or any of the eight languages to which all the information materials and questionnaires were translated (Arabic, English, Sorani, Somali, Tamile, Turkish, Urdu and Vietnamese), and be able to give informed written consent. Exclusion criteria were pre-pregnancy diabetes or other diseases requiring intensive hospital follow-up during pregnancy, and participation in the same study during a previous pregnancy lasting ≥22 weeks.

A total of 823 women were recruited to the study between May 2008 and May 2010. Participation rate was 74% among all eligible women and varied by ethnicity (European 82%, South Asian 73%, Middle Eastern 65%, African 64% and East Asian 63%). The final sample size was 811 women after excluding 12 women from Central or South America due to small group size. This study was conducted according to the guidelines laid down in the Declaration of Helsinki and all procedures involving human subjects were approved by the Regional Ethics Committee and the Norwegian Data inspectorate. Written informed consent was obtained from all subjects.

### Data collection

#### Outcome variables

The main outcome variables were folic acid supplement use before and during pregnancy. These data were retrieved from hospital records. Women were asked questions on supplement use in an interview at the ultrasound screening in the hospital at 17 to 19 week’s gestation, as part of the routine report for the Medical Birth Registry of Norway. The two questions elicited whether or not the women had used folic acid containing supplements before or during pregnancy by the time of the visit. In one of the hospitals, only a “yes” option was included in the form and “no” was imputed for women with a missing value (concerns 16.6% of pre-pregnancy data and 13.7% of pregnancy data in the whole cohort). No data on the exact timing, dose or duration of use were obtained for the present study.

#### Ethnicity and background variables

At the inclusion visit at 15 weeks’ gestation on average, the study midwives interviewed the participants and filled in a questionnaire eliciting questions on ethnic origin and other background information [21]. When needed, professional interpreters were used. Ethnicity was defined based on the participant’s country of birth or her mother’s country of birth if the mother was born outside of Europe or North America. For the present study, ethnicity was categorized to five groups: Europe (including North America), Middle East (including North Africa and Central Asia), Africa (except for North Africa), South Asia and East Asia. European women were used as the reference group. By ethnicity, 82.6% of them were Norwegian, 3.4% Swedish or Danish, 2.6% other Western European (including three white North American women) and 11.3% Eastern European. Western and Eastern European women were first analyzed as separate groups, but since they had very similar folic acid supplement use and the number of Eastern European women was quite small (*n* = 43), the groups were merged in the final analyses.

Age and weeks’ gestation at the inclusion visit were used as continuous variables. Duration of residence in Norway was categorized to 0–1 years (recent immigrants) or ≥2 years for women not born in Norway. Norwegian language skills were elicited with five response options (poor, quite poor, average, quite good or good) and were further categorized to “poor or quite poor”, “average or quite good”, or “good” (the value “good” was imputed for all ethnic Norwegians). Educational level was categorized as “high-school or less” (i.e. less educated) or “college or university” (i.e. more educated) based on six original categories. Parity was categorized to “nulliparous” or “parous” women. The question on planned pregnancy had three response options (yes, no, partly) which were re-categorized to “yes” or “no” (including “partly”). By “partly” we mean “not using contraceptives, with a wish to become pregnant, although not planning to get pregnant at a specific time”.

### Statistical methods

Descriptive data are presented as means and standard deviations (sd) or numbers and percentages. Overall ethnic differences in the crude percentages of folic acid supplement use were tested using two-sided *χ* ^2^ –test, separately among all women and stratified by educational level and planning of pregnancy. Ethnic differences in supplement use before and during pregnancy (the dependent variables) were analyzed further by logistic regression models. Unadjusted analyses were performed for each covariate. Adjusted models were carried out using Enter-method, adjusting simultaneously for age, parity, planning of pregnancy and educational level (Model 1). Model 2 was additionally adjusted for Norwegian language skills to demonstrate how inclusion of language skills changes the estimates. These variables were chosen as covariates as they were associated with ethnicity in the present data and with folic acid supplement use in previous literature. We performed preliminary analyses in which socio-economic status score [22, 23] was used instead of education and the results were essentially the same. In the final models, education was used as an indicator of socio-economic status as they were strongly associated and educational level is easier to interpret than the socio-economic status score [22].

To test for effect modification between ethnicity and planning of pregnancy and between ethnicity and education, the ethnicity variable was re-categorized to ‘Europeans’ vs. ‘non-Europeans’ to increase statistical power in the analyses. The product terms ethnicity*planning of pregnancy and ethnicity*education were added to each regression model (Model 2) one by one. Planning of pregnancy did not modify the association between ethnicity and supplement use before or during pregnancy (*p* = 0.31 and *p* = 0.89, respectively, for the product terms). The product term for ethnicity*education was also not statistically significant for supplement use before pregnancy (*p* = 0.28). However, the product term was statistically significant for ethnicity and education when supplement use during pregnancy was the outcome (*p* = 0.013). Therefore, the adjusted Model 2 was also stratified by educational level. The results of the logistic regression models are presented as odds ratios (OR) with 95% confidence interval (CI), p-values and adjusted R^2^, and *p*-values <0.05 were considered as statistically significant. All analyses were conducted using the SPSS statistical software package version 23 (SPSS Inc., Chicago, IL, USA).

## Results

Background characteristics of the participants are described by ethnicity in Table 1. The mean age varied from 28 to 31 years between the ethnic groups. Women from Africa and East Asia were most likely to be recent immigrants and African women were least likely to have good Norwegian language skills. Higher educational level was least common among African and Middle Eastern women and most common among European women. The percentage of parous women was lower in European women than in the other ethnic groups. The percentage of women with planned present pregnancy was highest among European and lowest among East Asian women. African and East Asian women were included in the study at 17.3 and 16.3 weeks’ gestation, respectively, whereas European women were included already at 14.3 weeks’ gestation on average.

**Table 1:** Background characteristics of the participants by ethnicity, means (sd) or numbers (%)^a^

	Europe *n* = 379 (46.7%)		South Asia *n* = 200 (24.7%)		Middle East *n* = 126 (15.5%)		Africa *n* = 62 (7.6%)		East Asia *n* = 44 (5.4%)	
	n	Mean (sd) /%	n	Mean (sd) /%	n	Mean (sd) /%	n	Mean (sd) /%	n	Mean (sd) /%
Age (years)	379	30.2 (4.5)	200	28.2 (4.6)	126	29.0 (5.5)	62	27.7 (5.2)	44	30.8 (4.9)
Born in Norway, n (%)	299	78.9	41	20.5	7	5.6	1	1.6	1	2.3
Recent immigrants^b^, n (%)	8	12.3	17	10.7	14	12.0	14	23.0	9	20.9
Norwegian language skills, n (%)										
Poor or quite poor	8	2.1	34	17.0	29	23.2	16	25.8	11	25.0
Average or quite good	24	6.3	80	40.0	58	46.4	31	50.0	19	43.2
Good	346	91.5	86	43.0	38	30.4	15	24.2	14	31.8
Educational level, n (%)										
High school or less	133	35.4	136	68.3	101	81.5	54	87.1	27	61.4
College or university	243	64.6	63	31.7	23	18.5	8	12.9	17	38.6
Parity ≥1, n (%)	175	46.2	116	58.0	82	65.1	35	56.5	26	59.1
Planned pregnancy, n (%)	245	69.2	125	65.4	73	63.5	32	60.4	18	46.2
Weeks’ gestation at the inclusion visit	379	14.3 (2.4)	200	15.6 (3.9)	126	15.0 (3.3)	62	17.3 (4.9)	44	16.2 (3.9)

^a^A total of 59 women had a missing value for planned pregnancy and the numbers varied by ethnicity as follows: Europeans 25 (6.6% of all Europeans), South Asian 9 (4.5%), Middle Eastern 11 (8.7%), East Asian 5 (11.4%) and African 9 (14.5%). The total numbers of missing values for the other variables were: recent immigrants (*n* = 17 among women not born in Norway), educational level (*n* = 6), Norwegian skills (*n* = 2) and other variables (*n* = 0)

^b^<2 years’ residence in Norway among women who were not born in Norway

### Folic acid supplement use before pregnancy

**Fig. 1:**
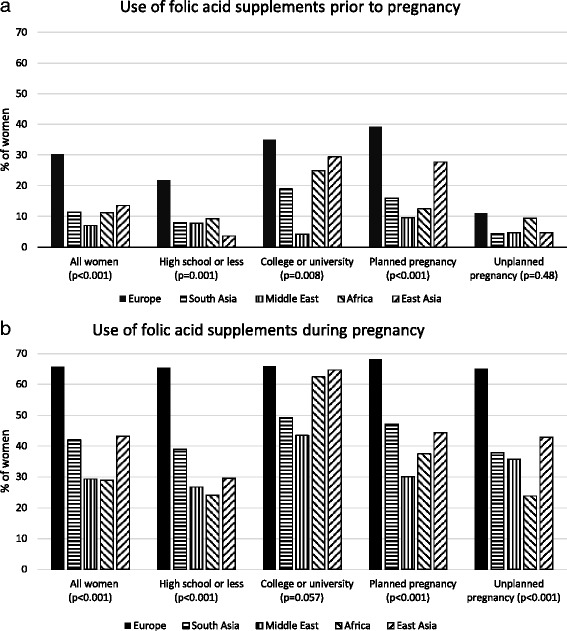
**a** and **b**. Ethnic differences in folic acid supplement use before and during pregnancy. Among all women and stratified by educational level and planning of pregnancy, crude percentages

The use of folic acid supplements before pregnancy was uncommon in all ethnic groups, but European women (30.1%) used the supplements more often than the other ethnic groups (7.1 to 13.6%) (Fig. 1a). When stratifying by educational level, similar ethnic differences were observed at both levels. Supplement use seemed to be more common among more educated than among less educated women in most ethnic groups. When stratified by planning of pregnancy, European women were most likely and Middle Eastern and African women were least likely to have used the supplements before pregnancy among women with a planned pregnancy. However, no statistically significant differences were observed in supplement use between ethnic groups among women with an unplanned pregnancy.

Compared with European women, all other ethnic groups had decreased odds for using the supplements in the unadjusted logistic regressions (Table 2). Lower age, being parous, not planning pregnancy, lower educational level and poorer Norwegian language skills were also associated with not using the supplements before pregnancy. Adjustment for these variables attenuated the ethnic differences in supplement use. In Model 1, lower odds for using the supplements were observed among Middle Eastern (OR 0.26) and South Asian women (OR 0.40). In Model 2, when further adjusted for Norwegian language skills, the same ethnic groups differed from the reference group, and being parous, not planning pregnancy and poorer Norwegian language skills remained independently associated with not using the supplements. Of all 159 women who started using folic acid supplements before pregnancy, 156 (98%) continued using them during pregnancy.

**Table 2:** Logistic regression models for the use of folic acid supplements before pregnancy^a^

	Supplement users, n (%)	Unadjusted models		Adjusted Model 1		Adjusted Model 2	
				*n* = 749, *R* ^*2*^ = 0.22		*n* = 749, *R* ^*2*^ = 0.22	
		OR (95% CI)	*p*	OR (95% CI)	*p*	OR (95% CI)	*p*
Ethnicity							
Europe	114 (30.1)	Reference	Reference			Reference	
South Asia	23 (11.5)	**0.30 (0.19; 0.49)**	**<0.001**	**0.40 (0.23; 0.67)**	**0.001**	**0.55 (0.31; 0.96)**	**0.037**
Middle East	9 (7.1)	**0.18 (0.09; 0.37)**	**<0.001**	**0.26 (0.12; 0.56)**	**0.001**	**0.42 (0.19; 0.94)**	**0.034**
Africa	7 (11.3)	**0.30 (0.13; 0.67)**	**0.003**	0.42 (0.17; 1.08)	0.072	0.71 (0.26; 1.93)	0.50
East Asia	6 (13.6)	**0.37 (0.15; 0.89)**	**0.027**	0.60 (0.23; 1.53)	0.28	1.02 (0.38; 2.74)	0.98
Age, years	-	**1.04 (1.001; 1.08)**	**0.046**	1.01 (0.97; 1.06)	0.62	1.02 (0.97; 1.07)	0.43
Parity							
0	95 (25.2)	Reference		Reference		Reference	
≥ 1	64 (14.7)	**0.51 (0.36; 0.73)**	**<0.001**	**0.65 (0.43; 0.98)**	**0.04**	**0.62 (0.40; 0.94)**	**0.023**
Planned pregnancy							
No	20 (7.7)	**0.23 (0.14; 0.38)**	**<0.001**	**0.26 (0.16; 0.44)**	**<0.001**	**0.26 (0.15; 0.43)**	**<0.001**
Yes	132 (26.8)	Reference		Reference		Reference	
Educational level							
High school or less	54 (12.0)	**0.32 (0.22; 0.46)**	**<0.001**	**0.60 (0.38; 0.93)**	**0.021**	0.65 (0.42; 1.02)	0.058
College or university	105 (29.7)	Reference		Reference		Reference	
Norwegian language skills							
Poor or quite poor	5 (5.1)	**0.15 (0.06; 0.36)**	**<0.001**			**0.26 (0.10; 0.72)**	**0.009**
Average or quite good	19 (9.0)	**0.27 (0.16; 0.44)**	**<0.001**			**0.46 (0.25; 0.83)**	**0.010**
Good	135 (27.1)	Reference				Reference	

^a^Unadjusted models were performed separately for each variable in the Table. Adjusted Model 1 included ethnicity, age, parity, planning of pregnancy and educational level. Adjusted Model 2 additionally included Norwegian language skills. Statistically significant findings (*p* < 0.05) are highlighted with bold

### Folic acid supplement use during pregnancy

The prevalence of using folic acid supplements during pregnancy was highest among European women (65.7%) and lowest among African (29.0%) and Middle Eastern women (29.4%) (Fig. 1b). Stratification by education showed that these ethnic differences were statistically significant among less educated women only. Lower education was related to not taking supplements among all other ethnic groups, but not among Europeans. When stratifying by planning of pregnancy, European women were more likely to have used the supplements than all other ethnic groups regardless of whether or not the pregnancy had been planned.

Compared to European women, all other ethnic groups had lower odds for supplement use during pregnancy in the unadjusted model and Model 1 (Table 3). In Model 2, the ORs were somewhat closer to one, but still different from the reference group except for the East Asian women. Lower education and poorer language skills were also associated with not using the supplements. When Model 2 was stratified by education due to effect modification, each ethnic minority group and women with poorer Norwegian language skills had lower odds for supplement use among the less educated women only. No ethnic differences were observed in supplement use among the more educated women, but age was inversely associated with use.

**Table 3:** Logistic regression models for the use of folic acid supplements during pregnancy^a^

	Supplement users, n (%)	Unadjusted models		Adjusted Model 1		Adjusted Model 2		Adjusted Model 2, women with high school or less		Adjusted Model 2, women with college or university	
				*n* = 749, *R* ^*2*^ = 0.14		*n* = 749, *R* ^*2*^ = 0.17		*n* = 419, adj. *R* ^*2*^ = 0.20		*n* = 330, adj. *R* ^*2*^ = 0.09	
		OR (95% CI)	*p*	OR (95% CI)	*p*	OR (95% CI)	*p*	OR (95% CI)	*p*	OR (95% CI)	*p*
Ethnicity											
Europe	249 (65.7)	Reference		Reference	Reference			Reference		Reference	
South Asia	84 (42.0)	**0.38 (0.27; 0.54)**	**<0.001**	**0.42 (0.28; 0.61)**	**<0.001**	**0.59 (0.39; 0.90)**	**0.015**	**0.50 (0.28; 0.90)**	**0.020**	0.60 (0.31; 1.19)	0.14
Middle East	37 (29.4)	**0.22 (0.14; 0.34)**	**<0.001**	**0.28 (0.22; 0.87)**	**<0.001**	**0.43 (0.25; 0.73)**	**0.002**	**0.32 (0.17; 0.61)**	**0.001**	0.71 (0.25; 2.00)	0.51
Africa	18 (29.0)	**0.21 (0.12; 0.39)**	**<0.001**	**0.26 (0.14; 0.50)**	**<0.001**	**0.42 (0.21; 0.84)**	**0.014**	**0.31 (0.14; 0.69)**	**0.004**	1.56 (0.25; 9.82)	0.63
East Asia	19 (43.2)	**0.40 (0.21; 0.75)**	**0.004**	**0.44 (0.22; 0.87)**	**0.018**	0.69 (0.33; 1.44)	0.32	**0.30 (0.10; 0.86)**	**0.025**	1.80 (0.53; 6.04)	0.35
Age, years	-	0.99 (0.96; 1.02)	0.40	**0.96 (0.92; 0.99)**	**0.019**	**0.96 (0.93; 0.998)**	**0.037**	0.97 (0.93; 1.02)	0.28	**0.94 (0.88; 0.996)**	**0.037**
Parity											
0	214 (56.8)	Reference		Reference		Reference		Reference		Reference	
≥ 1	193 (44.5)	**0.61 (0.46; 0.81)**	**<0.001**	0.82 (0.59; 1.15)	0.25	0.80 (0.57; 1.12)	0.20	0.92 (0.57; 1.49)	0.73	0.65 (0.40; 1.07)	0.093
Planned pregnancy											
No	125 (48.3)	0.78 (0.58; 1.06)	0.11	0.88 (0.63; 1.22)	0.44	0.90 (0.64; 1.25)	0.51	0.93 (0.60; 1.44)	0.74	0.84 (0.50; 1.44)	0.53
Yes	268 (54.4)	Reference		Reference		Reference		Reference		Reference	
Educational level											
High school or less	188 (41.7)	**0.45 (0.34; 0.60)**	**<0.001**	**0.64 (0.45; 0.91)**	**0.012**	**0.70 (0.49; 1.000)**	**0.050**				
College or university	217 (61.3)	Reference		Reference		Reference					
Norwegian language skills											
Poor or quite poor	23 (23.5)	**0.18 (0.11; 0.30)**	**<0.001**			**0.30 (0.17; 0.54)**	**<0.001**	**0.31 (0.16; 0.59)**	**<0.001**	0.28 (0.07; 1.12)	0.072
Average or quite good	72 (34.0)	**0.31 (0.22; 0.43)**	**<0.001**			**0.51 (0.34; 0.76)**	**0.001**	**0.42 (0.25; 0.71)**	**0.001**	0.62 (0.32; 1.22)	0.17
Good	312 (62.5)	Reference				Reference		Reference		Reference	

^a^ By 17 to 19 weeks’ gestation. Unadjusted models were performed separately for each variable in the Table. Adjusted Model 1 included ethnicity, age, parity, planning of pregnancy and educational level. Adjusted Model 2 additionally included Norwegian language skills. Statistically significant findings (*p* < 0.05) are highlighted with bold

## Discussion

This is the first study to report peri-conceptional folic acid supplement use in specified ethnic groups in Scandinavian countries. Few women used folic acid supplements before pregnancy and the use was particularly low among women of non-European origin. After adjusting for planning of the pregnancy, education, Norwegian language skills, and parity, Middle Eastern and South Asian women were still less likely to use the supplements before pregnancy than European women. The main finding was that association between ethnicity and folic acid supplement use during pregnancy was strongly modified by educational level. While all non-European ethnic minority groups had lower odds for supplement use during pregnancy among the less educated women, no ethnic differences were observed among the more educated women after adjusting for confounders.

Our results are mainly consistent with previous studies on ethnic differences in folic acid supplement use before and/or during pregnancy in Europe. Several studies used a dichotomous categorization of ethnicity and reported lower use of supplements among non-Western than among Western women [12, 14, 17–19]. A comparison of our results with a previous study in Oslo [12] from year 2001 shows that ethnic differences in supplement use have persisted, but the prevalence of use have slightly increased especially among ethnic minority women, both before and during pregnancy. Similar to our results, large studies from the Netherlands [13] and Ireland [15] comparing several ethnic groups showed that Middle Eastern and African women were less likely to use the supplements than Western or Western European women. In Ireland, women from Eastern Europe and South America also used less supplements than Western European women [15]. A very large study (*n* = 466860) from the United Kingdom found that South Asian, Oriental (East Asian) and Afro-Caribbean women were less likely to use folic acid supplement before pregnancy than Caucasian women [20]. A smaller British study (*n* = 402) found that supplement use was less common in West Indian, African and Asian women than in Caucasian women [16]. The prevalence of use was generally lower before pregnancy but higher during pregnancy than in our study. The other studies did not report comparable data [13, 15]. Our study was the second to analyze South Asians and East Asians as separate groups. It is noteworthy that all the included non-Western ethnic subgroups had lower use of the supplements than the Western reference group in these studies [13, 15, 16, 20]. However, the results of the other studies are not directly comparable to our results due to partly different ethnic groups and adjustment for different confounders.

Our study is the first to describe effect modification between ethnicity and other covariates in relation to folic acid supplement use. We observed effect modification between ethnicity and education and it remained statistically significant in the adjusted models. Only the less educated ethnic minority women were significantly less likely to use supplements during pregnancy than European women. Proficiency in the Norwegian language also played a role in non-European women, suggesting that the less educated non-European women may not have known the importance of using the supplements in early pregnancy due to language barriers. More educated non-European women may have found the information from other sources regardless of their language skills. One study suggests that the folic acid supplement use could be more strongly related to proficiency in the local language than ethnicity as such [13]. The study found that proficiency in Dutch was the strongest determinant of knowledge on the importance of folic acid supplement use during the peri-conceptional period and knowledge was the strongest determinant of actual use. Educational level also predicted knowledge on folic acid supplements. Higher education has been related to the use of folic acid supplements also in other studies [14, 17, 19].

Although planning of pregnancy modified the association between ethnicity and crude prevalence of folic acid supplement use before pregnancy (Fig. 1a), no statistically significant effect modification was observed in the adjusted models. However, planning of pregnancy was strongly associated with supplement use before pregnancy regardless of ethnicity, which has also been observed in some other studies [11, 14, 15, 24] but not in all [13]. Interestingly, parous women were less likely to use folic acid supplements than nulliparous women both in our study and several previous studies [11, 13–15, 18, 19, 24] although parous women should have heard about the benefits of supplement use during their previous pregnancy. Women who have previously delivered healthy children might trivialize the risks and be reluctant to follow recommendations on healthy behavior, as discussed by van Eijsden [13].

The strengths of our study include the population-based cohort design, the high proportion of ethnic minority groups and the relatively high, although not equal, participation rates in each ethnic group. The population was found to be representative of the main ethnic groups in Oslo, decreasing the likelihood of selection bias [21]. The data on supplement use were collected by mid-pregnancy when women were more likely to remember possible supplement use than if the data were collected later in pregnancy. However, we do not know when the women started using supplements and if they stopped using them by 12 weeks’ gestation as recommended. Other possible limitations were that we have no data on subjective reasons for nonuse, for example unawareness on the importance of folic acid supplements. We cannot exclude the possibility that women with poor Norwegian language skills might have misunderstood the questions on supplement use and, although obligatory, translators may not always have been used at the hospitals, leading to a potential information bias. Additionally, “no” was imputed for women with missing data on supplement use from one of the participating hospitals (14 to 17% of the total population). However, this has probably not introduced a bias since the results were essentially similar when we used raw hospital data in which missing values were not coded as “no”. A total of 62 women had missing data on planned pregnancy or another variable and were therefore not included in the adjusted models. The percentages of missing values for planned pregnancy varied slightly by ethnicity (Table 1) and women with missing values might be different from the other women. We also categorized education into two broad categories as few Europeans had really low education. Therefore, within the category of “less educated women”, 89% of the European and 63% of the non-European women had finished high school and 14% of the non-European women had ≤7 years of education only. Low numbers in some ethnic groups also reduced statistical power especially in the stratified analyses.

Our findings have several implications for clinical and public health practice. Firstly, ethnic minority women of childbearing-age need information on the importance of using folic acid supplements before and during pregnancy. Secondly, while proficiency in local language is very important and should be promoted among immigrants, the information should also be available to women who cannot read or understand the local language. The information could at least be translated into several major languages. Thirdly, since a large proportion of pregnancies are not planned and also a minority of European women use the supplements before pregnancy, public health messages to use folic acid supplements should be aimed at all women of childbearing age who may become pregnant. The information could be disseminated for example on websites providing official health information and recommendations for pregnant women, at school health education classes, in student health care or via mass media campaigns. Fourthly, parous women should be reminded during late antenatal or postpartum visits about the importance of folic acid supplement use before possible subsequent pregnancies. Additionally, a potential, but controversial option would be to consider food fortification with folate as some authors have suggested [9].

## Conclusions

Pre-pregnancy use of folic acid supplements was uncommon in general and substantially lower among Middle Eastern and South Asian women as compared to European women, when adjusted for several confounders. Educational level strongly modified the association between ethnicity and folic acid supplement use during pregnancy. Among less educated women, all non-European ethnic groups were less likely to use folic acid supplements during pregnancy than European women. No significant ethnic differences were observed in women with higher education. Although awareness of the importance of folic acid supplement use should be increased among all women of childbearing age, ethnic minority groups need special attention.

## Abbreviations

CI: Confidence interval
OR: Odds ratio
Sd: Standard deviation
